# Robotic Pediatric Urologic Surgery-Clinical Anesthetic Considerations: A Comprehensive Review

**DOI:** 10.5812/aapm-146438

**Published:** 2024-06-18

**Authors:** Nazih Khater, Seth Swinney, Joseph Fitz-Gerald, Ahmad S. Abdelrazek, Natalie M. Domingue, Sahar Shekoohi, Farnad Imani, Tahereh Chavoshi, Ali Moshki, Kimberly L. Skidmore, Alan D. Kaye

**Affiliations:** 1 Department of Urology, Louisiana State University Health Sciences Center, Shreveport, LA, USA; 2 Department of Urology, Mayo Clinic, Rochester, MN, USA; 3 School of Medicine, Louisiana State University Health Sciences Center, Shreveport, LA, USA; 4 Department of Anesthesiology, Louisiana State University Health Sciences Center, Shreveport, LA, USA; 5 Pain Research Center, Department of Anesthesiology and Pain Medicine, School of Medicine, Iran University of Medical Sciences, Tehran, Iran

**Keywords:** Robotics, Pediatric Urology, Anesthesia, Outcomes, Physiology, Safety

## Abstract

Minimally invasive robotic approaches have become standard in many institutions over the last decade for various pediatric urological procedures. The anesthetic considerations for common laparoscopic and robotic-assisted surgeries are similar since both require the insufflation of CO_2_ to adequately visualize the operative area. However, few studies exist regarding anesthesia for robotic procedures in children. We hypothesized that pediatric patients undergoing robotic urologic surgeries would require specific anesthetic strategies, especially given the inherently longer durations of these procedures. This study aimed to evaluate anesthetic considerations, current robotic procedures, optimal patient positioning, and port placement for robotic-assisted surgery in pediatric patients. A comprehensive literature review of all published manuscripts from PubMed, EMBASE database, and Google Scholar was performed, focusing on robotic procedures involving pediatric patients, anesthesia for pediatric urology patients, and related topics from 1996 to 2023. Forty published manuscripts were identified and reviewed in depth.

In pediatric cases, insufflation pressures and volumes are lower due to the laxity of the abdominal wall. However, the increase in intra-abdominal pressure and absorption of CO_2_ may result in disproportionate changes in cardiopulmonary function. Specific patient positioning for robotic approaches may further compound these physiological changes. Correct patient positioning is essential to facilitate surgery optimally and safely. Understanding the physiological changes that can occur during a pediatric patient’s robotic urologic surgery allows for safer anesthesia management.

## 1. Context

Robotic pediatric urologic surgery has become ubiquitous in the last two decades, applied in a broader spectrum of procedures (1). Today, with the advancement of robotic devices, conventional pediatric urological procedures such as ureteral reimplantation and pyeloplasty are routinely performed, whereas in the past, they presented many technical challenges. Over the past 10 years, pediatric urologists have adopted robotic approaches for many procedures in children 18 years old or younger (2). It is crucial for practitioners to be familiar with the specific challenges of these techniques and know how to prepare an effective and safe anesthetic plan.

Although several published papers evaluate robotic laparoscopic procedures for urological interventions in the pediatric population, few have described anesthetic management strategies. To maximize the safety and efficacy of anesthetic measures for these surgeries, it is essential to have a comprehensive understanding of the physiological changes that occur during laparoscopic procedures in pediatric patients, as well as the possible adverse effects associated with this modality, especially considering that attaching the robot adds time to laparoscopic surgeries. A logical strategy for perioperative considerations for anesthesia and pain management optimizes outcomes.

While laparoscopic methods result in smaller scar tissue formation, fewer adhesion bands, decreased postoperative pain, and shorter hospital stays, several factors make anesthesia management challenging in these operations, particularly in younger children (3-5). This review highlights the advances in robotic pediatric urologic surgery and investigates how to formulate anesthetic strategies corresponding to each physiological derangement unique to children.

## 2. Methods

A comprehensive literature review of all published manuscripts was conducted by searching for keywords in PubMed, EMBASE database, and Google Scholar. Keywords included "robotic pediatric surgery," "anesthesia for pediatric urology," and related topics in articles published between 1996 and 2023. Forty published manuscripts were identified and reviewed in depth. Studies were selected to provide a comprehensive overview of robotic surgery in pediatric urology, including surgical considerations, anesthetic considerations for patients younger than 18 undergoing robotic procedures, and the types of robotic pediatric urologic procedures being performed.

## 3. Results

### 3.1. Pediatric Urology Surgical Considerations

Laparoscopic surgery has well-documented advantages over open surgery. Robotic-assisted surgery offers even more benefits, such as better visualization of tissue planes, stability of instruments, and improved precision of suturing for the operating surgeon. These factors explain the surge in pediatric urologic surgeries (6). The robotic approach to pediatric urologic procedures has evolved to include both lower and upper urinary tract surgeries (7, 8). Minimally invasive surgery (MIS) often requires the distension of the peritoneal cavity, achieved by insufflation of carbon dioxide (9). The compliance of the abdominal wall is greater, and the size of the peritoneal cavity is smaller in infants than in adults, therefore, less insufflation pressure (4-12 mmHg) is usually needed for visualization of intraperitoneal structures (10). Laparoscope (camera) insertion after expansion of the abdominal wall allows for the insertion of ports and surgical instruments.

The da Vinci^®^ surgical system has two key features: A tower with suspended arms and a chair with a video console. Robotic devices (e.g., needle holders, graspers, and cautery) are exchangeable inside the device arms. The usual movements of the surgeon’s hands are more similar to those in open surgery compared to laparoscopic surgery, thanks to the development of EndoWrist^®^. The robotic procedure is performed transperitoneally in most pediatric patients.

### 3.2. Anesthetic Considerations

Laparoscopic surgery has well-documented advantages over open surgery. Robotic-assisted surgery offers even more benefits, such as better visualization of tissue planes, instrument stability, and improved precision in suturing for the operating surgeon. These factors explain the surge in pediatric urologic surgeries (6). The robotic approach to pediatric urologic procedures has evolved to include both lower and upper urinary tract surgeries (7, 8). Minimally invasive surgery often requires the distension of the peritoneal cavity, achieved by insufflation of carbon dioxide (9). The compliance of the abdominal wall is greater and the size of the peritoneal cavity is smaller in infants than in adults, therefore, less insufflation pressure (4 - 12 mmHg) is usually needed for visualization of intraperitoneal structures (10). Laparoscope (camera) insertion after expansion of the abdominal wall allows for the insertion of ports and surgical instruments.

The da Vinci^®^ surgical system has two key features: a tower with suspended arms and a chair with a video console. Robotic devices (e.g., needle holders, graspers, and cautery) are exchangeable inside the device arms. The usual movements of the surgeon’s hands are more similar to those in open surgery compared to laparoscopic surgery, thanks to the development of EndoWrist^®^. The robotic procedure is performed transperitoneally in most pediatric patients.

### 3.3. Anesthetic Considerations

Although the choice of anesthetic agents for induction and maintenance may depend upon comorbidities, in otherwise healthy children, the low solubility inhaled anesthetic sevoflurane is titrated to one minimum alveolar concentration (MAC) because it has a short duration of action. Sometimes a small dose of dexmedetomidine is added for preoperative anxiolysis or emergence delirium. Alternatively, sevoflurane is combined with a propofol infusion to evoke a Bispectral Index (BIS) of 40 – 50. Intraoperative nitrous oxide should be eliminated to minimize the hazard of intestinal dilatation (11, 12).

A Salem Sump orally inserted tube is placed to suction the stomach, and a Foley catheter is inserted to decompress the bladder. These are necessary to reduce the hazard of organ damage when inserting the needle for insufflation.

It is preferable to use a tracheal tube with a cuff even in younger infants, because elevation of intra-abdominal pressure (IAP) can impede ventilation. Muscle relaxants are usually used if needed for adequate mechanical ventilation and to prevent damage caused by patient movement. The administration of muscle relaxants is particularly necessary during the insertion of all robotic trocars at the start of the surgery, but after insufflation, it is less important, especially when continuous end-tidal sevoflurane MAC or BIS monitoring is used to assure some muscle relaxation. It is crucial to have atropine immediately available in a syringe with a needle, in case of severe bradycardia due to vagal responses related to insufflation.

There are no practice guidelines or statements from professional societies about anesthetic protocols for robotic urologic surgery in this population. Standard pediatric anesthesia is considered for induction and maintenance. Recommendations for fasting are published by the American Society of Anesthesiologists (ASA). Children should be permitted to receive unlimited clear liquids no later than two hours, breast milk four hours, and infant formula six hours prior to the induction of anesthesia (13). According to the ASA, as for any surgery, blood pressure is rechecked every 5 minutes, oxygen saturation is continuously audible by pulse oximeters, capnography is always present on the ventilator, electrocardiography of one lead is always present, and temperature is recorded if the anesthetic is prolonged. Additional monitoring (e.g., invasive intra-arterial pressure) needs to be selected depending on expected large blood loss, comorbidities, and duration of surgery. For most children, judicious anxiolytics are administered prior to the induction of anesthesia. These calm the child while avoiding respiratory depression (14).

For anxiolysis, the drug of choice is midazolam, which can be administered orally (0.3 – 0.7 mg/kg, not to exceed 20 mg), nasally (0.3 mg/kg), or intramuscularly (0.3 mg/kg) (15). If a child remains uncooperative and unmanageable, intramuscular ketamine doses of 4 - 5 mg/kg are considered appropriate for sedation in most cases, up to 100%. However, as long as bradycardia is not a problem, dexmedetomidine nasally (0.2 mcg/kg) may be trialed first. Anticholinergic agents can be administered to avoid reflex bradycardia following insufflation (e.g., atropine or glycopyrrolate). Induction can begin with either intravenous or inhalational agents, depending on the child’s tolerance to the insertion of an intravenous catheter. For practical reasons, inhalational induction is favored in younger children. Compared to inhaled induction, intravenous induction is more rapid. Propofol is the best intravenous agent since it reduces movement during intubation and provokes less nausea than sevoflurane. The preferred agent for inhaled induction is usually sevoflurane due to its least pungent nature (in terms of airway secretions and bronchospastic reactivity) compared to desflurane and isoflurane. Inhalational induction should not be done with desflurane because it stimulates coughing, secretions, and laryngospasm (16).

Regarding airway management specific to robotic urological procedures, endotracheal intubation is superior to the supraglottic airway (SGA) (17). An endotracheal tube controls ventilation and prevents aspiration (18). Standard instructions about precautions that may reduce the risk of subglottic stenosis have urged the use of uncuffed endotracheal tubes (ETT) if the child is 4 years old or younger, but a leak around an uncuffed ETT can prevent adequate ventilation. Application of a cuffed tracheal tube will permit the use of higher peak airway pressure during pneumoperitoneum. Therefore, ETT intubation with minimum cuff inflation (to the level of an audible leak at approximately 25 CmH_20_ peak pressure, or measured by a pressure gauge on the pilot balloon) can balance the risks of ischemia at the tracheal mucosa with difficulties in ventilation (19). The use of SGAs during robotic surgery in children is controversial (18), although it has been safely used for short-term surgeries (20). Intraoperative maintenance of anesthesia for robotic surgery may involve primarily intravenous or inhalational agents, as is commonplace for open abdominal surgery. The anesthetic protocol is completed by intravenous narcotics such as remifentanil or fentanyl.

Intraoperative muscle relaxants are typically administered to facilitate tracheal intubation and optimize surgical conditions, but the published literature regarding the desirable levels of muscle relaxation during laparoscopic procedures is controversial (21).

Most pediatric patients require controlled ventilation. The new generation of ventilators can deliver small tidal volumes slowly, enabling lung-protective strategies for pediatric patients. Although evidence in pediatric surgical settings linking ventilation volumes to outcomes is limited, lung-protective ventilation should be considered at least as important as it is for adults. The protocol aims to maintain a tidal volume between 6 to 7 mL/kg, with judicious use of PEEP to prevent atelectasis. Recruitment maneuvers, such as holding 20 cmH_20_ PEEP for five seconds manually, can reverse suspected instances of atelectasis (22). Implementing such a protocol requires a ventilation mode that allows monitoring of the interaction between the patient and the ventilator. This can be challenging due to the difficulty in matching exact weight to monitor readings of exhaled tidal volume in pediatric patients, where the compliance of the tubing in the ventilator circuit can be confounding. Efficiency is maximized with a pressure-controlled mode, as it provides the highest inspiratory flow rate available among the limited modes on anesthesia ventilators. A cloth bite guard can prevent kinking of the ETT between the teeth. Small ETT suction catheters (6, 8, 10 French for 3, 3.5, 4 mm ETT, respectively) must be available to clear mucus plugs. The ETT tends to migrate into the right main stem bronchus more frequently in children than in adults, with an incidence of approximately 25% after insufflation when IAP increases, pushing the abdominal contents cephalad. The most accurate method to ensure the ETT is not in the mainstem bronchus is palpation of the pilot balloon while ballottement of the cuff is performed at the suprasternal notch. In summary, the anesthetist empirically chooses the optimal ventilation strategy, adjusting in real time to correct gas exchange.

Goals include maintaining arterial oxygen saturation above 90% at the lowest possible inspired oxygen fraction, ensuring acceptable arterial CO_2_ tension as estimated by end-tidal CO_2_, and achieving exhaled tidal volumes of 7 mL/kg at the lowest possible inspiratory pressure. Another factor to monitor is fluid status. Perioperative fluid requirements depend upon factors such as dehydration, age, vascular resistance, and the interventions, where robotic procedures are associated with less third spacing and blood loss than open procedures. The most important goal is to maintain euvolemia (23, 24). Robotic-assisted surgeries are a risk factor for postoperative nausea and vomiting; for this reason, prophylaxis by dual antiemetics should be given to all patients (25). The pain score after robotic procedures is often less than after open procedures, but the level of pain depends on what type of procedures. The popular analgesics after robotic pediatric urologic surgeries are paracetamol, NSAIDs, or narcotics. In addition, local anesthetic infiltration into the incisions should be performed during wound closure (26). A summary of anesthetic considerations in robotic pediatric urologic surgery is presented in Box 1. 

**Box 1.** Anesthetic Considerations in Robotic Pediatric Urologic Surgery

Summary of Anesthetic Considerations in Robotic Pediatric Urologic Surgery
Combination of a shorter acting inhaled anesthetic (e.g. sevoflurane) and a low-dose propofol infusion titrated to a Bispectral Index (BIS) level of 40 – 50.
Two intravenous lines
A cuffed endotracheal tube is preferred.
Availability of atropine is mandatory in the occurrence of sever bradycardia.
When anxiolysis is required, midazolam is the drug of choice.
Utilization of a cuffed ETT can be beneficial in the application of PEEP and high peak pressure in the airways during pneumoperitoneum.
Ventilator parameters: Appropriate PaO_2_ at least FiO_2_, optimal PaCO_2_, delivered tidal volumes at least inspiratory pressure

### 3.4. Current Robotic Pediatric Urologic Procedures

Since FDA approval of robotic surgery in 2000, when robotic-assisted radical retropubic prostatectomy ushered in this hallmark first application (27), various robotic urologic procedures have evolved sequentially throughout the world (Table 1). 

**Table 1.** Current Most Common Robotic Pediatric Urologic Procedures

Current Robotic Pediatric Urologic Procedures
(1) Robotic pyeloplasty
(2) Robotic ureteral re-implantation
(3) Robotic appendicovesicostomy/bladder augmentation
(4) Robotic partial nephrectomy, radical nephrectomy and nephroureterectomy
(5) Robotic prostatectomy
(6) Robotic orchiopexy for undescended testis (UDT)
(7) Robotic surgery in pediatric urolithiasis

#### 3.4.1. Robotic Pyeloplasty

Robotic pyeloplasty has been gaining favor over the last two decades (28-30). Pyeloplasty is currently the most popular robotic surgery performed in pediatric urology. Benefits afforded to the surgeon by the robot, specifically in pyeloplasty, include tremor cancellation, 3D vision, and optimal working space despite a normally confined area. Initially, laparoscopic pyeloplasty was not chosen in most centers because of technical difficulty and a steep learning curve, especially in centers with relatively low volumes.

- Positioning: Patients are placed at the lateral edge of the table at a tilt of 45 degrees. The lower leg is bent at a 90-degree angle at the knee, and the upper leg is straight at the hip.

- Initial access is via Veress needle, or the open Hassan technique if the patient is an infant.

- CO_2_ insufflation target is 8 - 10 mmHg.

- A 5 mm optical trocar is placed infraumbilically.

- Port placement is in a straight line with at least 3 cm separation.

- 8 mm trocars will replace the 5 mm viewing trocar.

- HIDES trocar positioning: Infraumbilical port and two lower abdominal ports.

- Most patients are discharged within 24 hours if able to void and tolerate a diet.

- Financial: The increase in cost is approximately $3000.

#### 3.4.2. Robotic Ureteral Re-implantation

Robotic ureteral re-implantation in the pediatric population has become popular over the past few years (31). Open ureteral re-implantation has a success rate of over 94%. Endoscopic approaches are the least invasive but have variable cure rates, frequently under 70%.

- Positioning: Lithotomy allows cystoscopy; then the robot is docked between the legs. Side docking allows for a supine position.

- Port placement: An 8mm robotic camera port at the umbilicus (Hasson or Veress). Working ports are on either side of the umbilicus inferiorly. Working ports may be unnecessary (31).

Over a decade ago, a study showed that over 97% of cases of bilateral nerve-sparing RALUR (Robotic Assisted Laparoscopic Ureteral Re-implantation) are successful (32). There were no complications or instances of urinary retention. Other investigators showed over a 90% success rate for unilateral RALUR, but unfortunately, only about 72% for children with bilateral cases (33).

#### 3.4.3. Robotic Appendicovesicostomy/Bladder Augmentation

Open robotic appendicovesicostomy and bladder augmentation have proven to be efficient. Robotic surgery, however, is associated with more favorable cosmesis, shorter length of stay, and lower levels of pain (34). Patients who are candidates for this complex operation typically have reduced pulmonary reserve, which is critical during the postoperative phase of recovery due to kyphoscoliosis. Therefore, a minimally invasive approach is desirable.

A robotic approach is recommended for children aged 6 years and older without prior multiple abdominal procedures, severe spinal deformities, or severe underlying disorders that affect position and length of surgery.

- Positioning: Lithotomy for cystoscopy with bilateral stent placement. Next, dorsal lithotomy with 30 degrees of Trendelenburg to keep the small bowel out of the field. Knees are low-lying, and arms are tucked.

- Port placement: A 12 mm camera port supraumbilical improves access to the appendix and bowel. The Hasson technique is favored.

- Left arm port placed 8 cm lateral to the umbilicus.

- Right arm port placed 9 – 10 cm lateral to the umbilicus.

- Another port placed 7 – 8 cm lateral to the right arm port.

- A 12 mm assistant port placed in the left upper quadrant.

#### 3.4.4. Robotic Partial Nephrectomy, Radical Nephrectomy and Nephroureterectomy

Indications for robotic partial nephrectomy, radical nephrectomy, and nephroureterectomy in the pediatric population remain similar to those in adults, such as kidney tumors, anatomical anomalies, and nonfunctioning kidneys. Recently, a series of pediatric patients were studied after robotic nephrectomies (35). About one-third of these were partial nephrectomies, with approximately 18 minutes of warm ischemia time. Negative margins were achieved in all cases. Assistance was provided by an experienced adult minimally invasive urologic surgeon. Port placement resembles that in adults, although, depending on a small body habitus, ports may need to be placed midline.

- Positioning: Lateral decubitus.

- Port placement: A 12 mm camera port supraumbilical, and all remaining 8mm robotic ports are in a straight line, starting cephalad at the mid-clavicular line, around 1 - 2 finger breadths below the costal margin.

- In left-sided nephrectomies, the left arm port is the most cephalad one, just above the camera, and the right arm port is caudal to the camera.

- In right-sided nephrectomies, the right arm port is the most cephalad one, just above the camera, and the left arm port is caudal to the camera.

#### 3.4.5. Robotic Prostatectomy

There is a paucity of published robotic prostatectomy cases in the pediatric population. Only one well-documented case report was revealed in our search: A pediatric robotic prostatectomy and pelvic lymphadenectomy in a young patient with prostatic embryonal rhabdomyosarcoma that was refractory to several chemotherapy regimens (36).

- Positioning: Supine. Using the da Vinci^®^ surgical platform, a gentle Trendelenburg is applied.

- Port placement: A 12 mm supraumbilical (camera port) and the remaining 8 mm robotic ports are inserted lateral to it (right arm port and left arm port).

#### 3.4.6. Robotic Orchiopexy for Undescended Testis (UDT)

Robotic-assisted laparoscopic orchiopexy for undescended testicles has been recently well described (37). Robotic assistance has provided better outcomes for surgeons (ergonomically) and patients (post-operatively), because pure laparoscopic orchiopexy—which remains the gold standard for intra-abdominal testicles—can be technically challenging. A modified robotic-assisted laparoscopic one-stage Fowler-Stephens orchiopexy (FSO) begins with the mobilization of the blood vessels and ligation of the artery as cranially as possible, maintaining a wide flap of peritoneum between the vessels and vas deferens. This is achieved by employing the “Prentiss” maneuver (37). In this report, five infants underwent this modified technique with a median surgical duration of under 100 minutes. All cases were completed without conversion to a two-step (i.e., open) procedure, and there were no cases of testicular atrophy.

- Port placement: A 12 mm infraumbilical camera port is inserted with bilateral abdominal 8 mm working ports. A 10 mm scrotal port is also placed.

#### 3.4.7. Robotic Surgery in Pediatric Urolithiasis

Robotic surgery in pediatric urolithiasis has been a rare surgical approach, but the rate may soon rise (38). For unknown reasons, the incidence of urolithiasis in pediatric cases seems to be increasing around the globe. Available data on the application of robotic procedures for pediatric urolithiasis is limited. One study in the literature from 2007 shows the effective application of robotic pyelolithotomy in a few adolescent patients with a significant stone burden (39). Ureteroscopy, Shock Wave Lithotripsy, and Percutaneous Nephrolithotomy remain ideal prior to consideration of robotic-assisted surgery for kidney stones. There is evidence that robotic surgery in pediatric urolithiasis is a valid choice after failed attempts of endoscopies for large complex stone burdens or in the case of concomitant anatomical anomalies of the collecting system (38, 39).

#### 3.4.8. Robotic Urologic Surgery in Infants

Robotic urologic surgery in infants (less than 12 months old) is very rare and more challenging due to their small body habitus and the exclusive use of 5 mm trocar ports (40). There are concerns regarding smaller spaces and the inability to use robotic monopolar curved scissors. Among 220 patients analyzed by Kawal et al., patients ≤ 1 year of age comprised only 28.6% of their study sample and were limited to pyeloplasties (40). Flank positioning is made for these patients with appropriate padding, leaving the flank exposed in case of the need to convert to open surgery. Anesthetic dosages are tailored to infants and their corresponding weight. In 2017, Paradise et al. published a series of 20 robot-assisted laparoscopic infant pyeloplasties performed using 5-mm instruments only, with no conversions to open surgery. The patients' ages ranged from 2 to 9 months old, and the average operative time was around 2.5 hours (41). Infants should be well relaxed with optimal muscle paralysis during general anesthesia, allowing better workspace in this already confined space. It appears that infants require at least 3 mg/kg of succinylcholine to produce reliable conditions for intubation. The length of effectiveness (6 – 8 min) is similar to the regular 1 mg/kg intubating dose in adults (42). In 2015, Avery et al. published a multi-institutional study of infants undergoing robotic pyeloplasty, finding a 91% success rate for reduction or resolution of hydronephrosis and an 11% complication rate, similar to laparoscopic approaches (2). In 2022, Carsel et al. described their unique series of infants 6 months or younger who underwent robotic urologic surgery for several conditions (43). Patients  ≤ 6 months and  ≥ 4 kg were included; they underwent robotic pyeloplasty, ureteroureterostomy, heminephrectomy, and robotic nephrectomy, with the authors able to use 8 mm robotic trocars for all procedures. Finkelstein et al. demonstrated that robotic surgery in infants is feasible for pediatric urologic conditions and validated two measurements that would allow urologists to determine if patients are candidates: The distance between both anterior superior iliac spines (ASIS) should be > 13 cm and the puboxyphoid distance (PXD) should be > 15 cm (4).

### 3.5. Post-operative Considerations

Post-operative care after robotic surgery in the pediatric population is similar to that in adults. Early ambulation is encouraged, and pain regimens with minimal opioids are preferred. Meier et al. described the exclusion of opioids in infants after robotic pyeloplasty by using caudal analgesia (44). Twenty-four patients underwent robotic pyeloplasty by a single surgeon and received a caudal block at the end of the procedure, followed by a non-narcotic post-operative pain regimen. Among them, only four cases required a single dose of opioids in the recovery room, needing no further analgesic medication during or after discharge. In a recent study, the average length of stay was merely one day (44).

## 4. Conclusions

At present, both formal and robotic laparoscopic approaches are widely performed in pediatric procedures, including those in infancy and neonates. Due to the significant success rate of minimally invasive methods in adults, these techniques have been considered standard methods of operative therapy for many pediatric conditions and have proven to be effective and safe.

Careful attention needs to be paid to diagnose each pathophysiologic derangement that might be induced and how to remedy it. Recent results show that these minimally invasive methods for pediatric procedures result in smaller scar tissue formation, less discomfort and pain, fewer adhesion bands, better analgesia, and faster hospital discharge.

Providing effective and safe anesthetic management for pediatric patients undergoing these surgeries requires full familiarity and sufficient knowledge of the intense physiologic effects of pneumoperitoneum on vital organs such as the cerebrovascular, renal, respiratory, and cardiovascular systems, as well as the possible adverse effects of this procedure.

Furthermore, patient positioning, fluid therapy, and ventilation management are essential considerations for robotic laparoscopic procedures in pediatric patients.

Widespread and extensive abdominal pain following pneumoperitoneum has a high prevalence, and available papers continue to investigate the most useful and practical modalities to decrease or eliminate this pain. First discovered in 2008, a study in 2020 demonstrated that a series of five manual airway recruitment maneuvers at 30 cmH_2_O, with the final lasting 5 seconds, all performed after CO_2_ insufflation ceased, effectively reduced pain in women during laparoscopic surgery. The theory posited is that it ameliorates atelectasis and its associated post-operative coughing and splinting. These authors also injected dilute bupivacaine under the diaphragm, showing additional benefit (45).

In this comprehensive review, 40 published manuscripts were identified and reviewed in depth. Due to the smaller size of the abdomen and the flaccidity of the abdominal wall in the pediatric population, lower insufflation pressures and volumes are needed. The increase in intraabdominal pressure and absorption of CO_2_ can result in changes in cardiopulmonary function, and specific patient positioning for laparoscopic/robotic approaches may further compound these physiological changes. Correct patient positioning is essential to facilitate surgery optimally and safely. Understanding the physiological changes that can occur during a pediatric patient’s robotic urologic surgery allows for safer anesthesia. More studies are warranted to focus on best practice strategies to ensure the safest outcomes using robotic surgical techniques in pediatric urological surgery procedures.

## Data Availability

The dataset presented in the study is available on request from the corresponding author during submission or after publication.
